# Signaling and structures underpinning conducted vasodilation in human and porcine intramyocardial coronary arteries

**DOI:** 10.3389/fcvm.2022.980628

**Published:** 2022-08-12

**Authors:** Kim A. Dora, JinHeng Lin, Lyudmyla Borysova, Timea Beleznai, Michael Taggart, Raimondo Ascione, Christopher Garland

**Affiliations:** ^1^The Vascular Pharmacology Group, Department of Pharmacology, University of Oxford, Oxford, United Kingdom; ^2^Biosciences Institute, Newcastle University, Newcastle upon Tyne, United Kingdom; ^3^Bristol Heart Institute and Translational Biomedical Research Centre, University of Bristol, Bristol, United Kingdom

**Keywords:** human coronary arterioles, coronary microvascular function, myogenic tone, Ca^2+^ signaling, conducted vasodilation, endothelial cell, bradykinin, adenosine

## Abstract

**Background:**

Adequate blood flow into coronary micro-arteries is essential for myocardial function. Here we assess the mechanisms responsible for amplifying blood flow into myogenically-contracting human and porcine intramyocardial micro-arteries *ex vivo* using endothelium-dependent and -independent vasodilators.

**Methods:**

Human and porcine atrial and ventricular small intramyocardial coronary arteries (IMCAs) were studied with pressure myography and imaged using confocal microscopy and serial section/3-D reconstruction EM.

**Results:**

3D rendered ultrastructure images of human right atrial (RA-) IMCAs revealed extensive homo-and hetero-cellular contacts, including to longitudinally-arranged smooth muscle cells (l-SMCs) found between the endothelial cells (ECs) and radially-arranged medial SMCs (r-SMCs). Local and conducted vasodilatation followed focal application of bradykinin in both human and porcine RA-IMCAs, and relied on hyperpolarization of SMCs, but not nitric oxide. Bradykinin initiated asynchronous oscillations in endothelial cell Ca^2+^ in pressurized RA-IMCAs and, as previously shown in human RA-IMCAs, hyperpolarized porcine arteries. Immunolabelling showed small- and intermediate-conductance Ca^2+^-activated K^+^ channels (K_Ca_) present in the endothelium of both species, and concentration-dependent vasodilation to bradykinin followed activation of these K_Ca_ channels. Extensive electrical coupling was demonstrated between r-SMCs and l-SMCs, providing an additional pathway to facilitate the well-established myoendothelial coupling. Conducted dilation was still evident in a human RA-IMCA with poor myogenic tone, and heterocellular contacts were visible in the 3D reconstructed artery. Hyperpolarization and conducted vasodilation was also observed to adenosine which, in contrast to bradykinin, was sensitive to combined block of ATP-sensitive (K_ATP_) and inwardly rectifying (K_IR_) K^+^ channels.

**Conclusions:**

These data extend our understanding of the mechanisms that coordinate human coronary microvascular blood flow and the mechanistic overlap with porcine IMCAs. The unusual presence of l-SMCs provides an additional pathway for rapid intercellular signaling between cells of the coronary artery wall. Local and conducted vasodilation follow hyperpolarization of the ECs or SMCs, and contact-coupling between l-SMCs and r-SMCs likely facilitates this vasodilation.

## Introduction

Cardiac function is critically dependent on the effective autoregulation of blood flow through the myocardium, and is controlled by the small arteries and arterioles of the microcirculation (1, 2). However, routine assessment of the functional state of the microcirculation is difficult *in vivo*, highlighting the importance of unraveling intramyocardial coronary artery (IMCA) signaling mechanisms under defined conditions *ex-vivo* (3–6).

Blood flow autoregulation relies largely on metabolic modulation of the inherent spontaneous myogenic activity, or tone, of small arteries. To become effective, local vasodilation must spread over distance. This is achieved by the phenomenon of conducted vasodilation, **first** recognized in skeletal muscle (7, 8). Conducted vasodilation usually relies on the spread of hyperpolarizing current along the endothelium, away from the local site of initiation, then back to the adjacent smooth muscle via heterocellular, myoendothelial gap junctions. Hyperpolarization of the smooth muscle reduces the open probability of voltage-gated calcium channels (VGCCs) affecting vasodilation (9). It follows that to be effective, locally released vasodilator autacoids (for example in ischaemic regions) must stimulate smooth muscle hyperpolarization, either directly or indirectly *via* the endothelium.

The ability to sustain conducted vasodilation is poorly characterized in coronary arteries, to date shown in porcine sub-epicardial coronary arterioles and human intra-pectinate coronary arterioles (10, 11). In the latter, bradykinin initiated conducted vasodilation by activating endothelium-dependent hyperpolarization (EDH), with endothelial nitric oxide (NO) suggested to facilitate this conduction to facilitate this conduction (10). Bradykinin is an endogenous vasodilator in the coronary circulation and during vasoconstrictor-induced tone in human coronary arterioles stimulates hyperpolarization by activating endothelial calcium-activated potassium channels (K_Ca_) (12, 13).

The present study was designed to extend the very limited data available regarding conducted vasodilation in myogenically contracting human and pig coronary IMCAs, probing whether similar underlying mechanisms operate. We also aimed to establish whether the intra-pectinate arteries routinely available from discarded human atrial appendage, and less available ventricular micro-arteries from organ donors, behave in a similar manner to equivalent porcine arteries. Our data link to previous studies with porcine ventricular sub-epicardial and sub-endocardial arterioles and large epicardial coronary arteries (11, 14–16) and indicate that porcine arteries provide an appropriate non-primate alternative to human arteries for cardiovascular studies (17).

## Methods

### Human and porcine study design and isolation of IMCAs

#### Human right atrial appendage biopsies

We used arteries from 45 patients (aged ≥25 and ≤80 years), 43 with valvular disease requiring either aortic valve replacement (AVR) or mitral valve repair/replacement (MVR) or both (AVR+MVR) and 3 with obstructive coronary disease, each undergoing elective or urgent cardiac surgery, a subset of patients from a previous study (18). These patients were chosen for this study based on their arteries developing >10% myogenic tone which was fully reversed with bradykinin, our previously defined inclusion criteria for viable arteries (18). Patient demographics and exclusion criteria are outlined in Table 1. We included data from two additional patients whose arteries had contractile dysfunction, for comparison purpose only, with the same exclusion criteria. The patient details are provided in Table 2. The University Hospital Bristol NHS Foundation Trust sponsored the study in Bristol, and the trial was approved by the Oxford Research Ethics Committee and extended to the Bristol site. The research complies with the Helsinki Declaration. RA biopsy samples from recruited patients were transported to Oxford under a material transfer agreement under strict packaging, temperature regulation (~10°C) and time-limit conditions.

**Table 1:** Patient demographics and exclusion criteria (*n* = 45)^*^.

			***n***	**%**
Sex	Female/male	17/28	45	
Age, yrs	mean ± SD	62 ± 12	45	
Surgical procedure	Valve repair/replacement		43	96
	CABG		3	7
Underlying risk factors	Hypertension		21	47
	Hypercholesterolemia		18	40
	Previous MI		4	9
	Decompensated CHF		1	2
	Large coronary artery disease		3	7
	Diabetes Mellitus		8	18
	Smoking History (<1 month)		4	9
	None of the above		18	40
Baseline medications	Statins		21	47
	Diuretics		11	24
	Anticoagulation		7	16
	Beta-blockers		14	31
	ACE-inhibitors		13	29
	Aspirin		12	27
Exclusion criteria	Age <25 years or >80 years			
	Pulmonary hypertension >50 mmHg			
	Impaired right ventricular function			
	Severely dilated atria (>5.0 cm)			
	Need for redo cardiac surgery			
	Need for ascending/root aortic surgery			
	Emergency surgery			
	Acute endocarditis			
	Infection, known HIV, Hepatitis A, B, C			
	Cancer or receiving chemotherapy			
	Immune disease			
	Ongoing pregnancy			

SD, standard deviation; CHF, Congestive Heart Failure; AVR, aortic valve replacement; MVR, mitral valve repair/replacement; CABG, coronary artery bypass grafting; HIV, human immuodeficiency virus. These data form a subset of 88 patients recruited for a full assessment of vascular reactivity (18).

* Two additional patients with the same exclusion criteria were included in this study for comparison purposes only, experimental data were not included in data summaries (patient demographics are provided in Table 2).

**Table 2:** Human RA-IMCAs processed for SBF-SEM arteries (*n* = 3).

**Artery**	**Patient demographics**	**MT (%)**	**Vasodilation to 10 nM BK (%)**	**Conducted dilation to BK (Y/N)**
#1	Female, 69 yrs, NT, NL, valve surgery; LV function good, >50% Meds: Statins	14.7	42.0	Y
#2*	Male, 52 yrs, HT, HL, CABG surgery; LV function poor, <30% Meds: Beta-blocker, ACE-inhibitor, diuretic, anticoagulant, statin	5.8	0.0	Y
#3*	Female, 81 yrs, HT, HL, valve surgery; LV function good, >50% Meds: ACE-inhibitor, diuretic, statin	0.0	0.0	-

In addition to one viable artery (Artery #1), arteries from two additional patients were isolated, cannulated and pressurized for assessment of vascular reactivity and then processed for SBF-SEM. Arteries #2 and #3 were not viable (MT <10%) and therefore data were not included in data summaries; they are shown for comparison purposes only. Data are presented in the Figures and Movies listed. MT, myogenic tone; BK, bradykinin; NT, normotensive; HT, requiring treatment for hypertension; NL, normal lipids; HL, requiring treatment for hypercholesterolaemia; Meds, baseline medications.

#### Human left ventricle biopsies from organ donors

Human heart tissue (Newcastle) was provided by The Newcastle Institute of Transplantation Tissue Biobank, the same patients as in our previous study (18). Once the biopsy was removed, the protocols matched the strict procedures used for the human cardiac surgery RA biopsies.

#### Porcine right atrial appendage and left ventricle biopsies

All the animal procedures were undertaken at the University of Bristol large animal facilities. All procedures were approved by University of Bristol Research Ethics Committee and performed in accordance with the Guide for the Care and Use of Laboratory Animals (19), the United Kingdom Animal (Scientific Procedures) Act, 1986, and conform to the guidelines from Directive of the European Parliament on the protection of animals used for scientific purposes. The animals recruited are the same as from our previous study (18). All animals were undergoing cardiac surgery with right atrial biopsies collected prior to cardiopulmonary bypass, and left ventricular biopsies from the endocardial surface collected at termination. All porcine biopsies were collected, packed and promptly couriered to Oxford using the same approach used for human RA and LV biopsies.

While arteries from each biopsy were all used in our previous study (18), each artery was subsequently used for additional protocols to form the new data presented here. The previously published values for myogenic tone and control dilation responses to bradykinin were necessarily included in this study.

Once in the laboratory, arteries were carefully cleared of surrounding tissue, avoiding side branches, and transferred to a myograph chamber (2 mL, RC-27 Warner Instruments, Hamden, CT) containing chilled MOPS buffer, held within the stage of an Olympus microscope, as previously described (18).

### Vascular reactivity of *ex vivo* pressurized IMCAs

#### Diameter studies

In all studies, IMCAs were mounted onto pipettes at each end with one end open to 80 mmHg (generated by gravity) and the other end closed to flow, meaning that luminal flow was effectively prevented [limited to leak across the ECs (18) and small changes during changes in diameter]. IMCAs were visualized using Olympus linescan confocal microscopes (FV300, FV500, FV1000 or FV1200). When studying function, arteries were imaged with transmitted light using a 10x Olympus objective and recorded using Fluoview software (Olympus, Tokyo, Japan) at 1 Hz, as previously described (18, 20). Myogenic tone was assessed as the tone developed in response to 80 mmHg luminal pressure, relative to the maximum diameter of arteries as previously described (18). Of the original study (*n* = 88 patients) which included many arteries with poor myogenic tone (*n* = 39), only arteries with >10% myogenic tone were used for this study (*n* = 45 of possible *n* = 49 patients). Endothelial cell (EC) function was assessed using bradykinin; arteries with >95% dilation to 1 nM and 1 μM bradykinin in porcine and human RA-IMCAs, respectively, were used. Values are the mean ± SEM of *n* patient or pig samples, one artery per sample unless otherwise stated.

*Cumulative concentration response curves* (CRCs) to bradykinin were obtained by addition of <20 μL doses of concentrated bradykinin (in MOPS buffer) to the edge of the warmed, static bath in a 2 mL imaging chamber, with gentle trituration to mix to the desired bath concentration, in 10-fold steps. The artery was exposed to each concentration for up to 120 s, which allowed peak responses to be recorded. Blockers were added to the superfusion flow for at least 20 min prior to commencing CRCs for dilator agonists, the vehicle not having any effect against MT or agonist responses.

*Conducted dilation* experiments were performed in arteries with one end closed to luminal flow to reduce the possible influence of shear stress. To limit agonist delivery to a confined region of the artery (termed downstream end), the superfusion flow rate longitudinally along the outside of arteries was near 2 mL/min. Bradykinin (10 μM or 1 μM for human or porcine IMCAs, respectively) or adenosine (100 μM) was delivered as a short bolus using a pressure or syringe pump positioned at the downstream end of the artery and near the imaging midplane of the artery (21). Note that this method of applying an agonist is akin to a bolus dose rather than steady state concentration. The bradykinin is diluted before reaching the lumen of the artery, and is delivered transiently, so the concentrations required to evoke dilation are higher than those when added to a static bath. Our previous studies have demonstrated that the direction of the superfusion flow does not always represent the direction of flow across the artery, due to the turbulence generated by the cannulating pipettes (21). Therefore, to ensure the delivery of agonists remained at the downstream end of the arteries, carboxyfluorescein (250 nM) was included in the pump micropipette to report the direction of agonist flow using confocal microscopy. The pump micropipette was moved to the other end of the artery as flow direction necessitated (21). This meant that we were able to visualize the delivery of agonists and subsequent conducted vasodilation across the entire artery assessing vasodilation at all points along the artery segment following a single, local application of the agonist. When using a 10x objective, >1,200 μm lengths of arteries were imaged in a field of view for simultaneous diameter and fluorescence measurements at positions 0–1,000 μm from the stimulating pipette. The default imaging field was *circa* 1,440 x 440 μm, at 1.4 μm/pixel. The delivery of agonist ceased as the artery dilated, which was within a few seconds. The same pipette position and delivery protocol was used for each given artery, with and without the addition of blockers to the superfusion solution. To compare the decay of dilation along each artery, data were normalized to the response at 200 μm from the delivery pipette, a site considered upstream to direct agonist delivery and more dependent on the spread of hyperpolarizing current. Thus the simultaneous values for diameter along the artery were obtained when the vasodilation to the agonist was 80% of maximal diameter at 200 μm, in a manner similar to previously (22, 23).

#### EC intracellular Ca^2+^ studies

After establishing the artery vasoreactivity, changes in arterial EC intracellular Ca^2+^ were imaged using the fluorescent Ca^2+^-indicators Oregon Green 488 BAPTA-1 or fluo-8 as previously (18). After excitation at 488 nm, fluorescence emission intensity was captured from at least 10 cells in the field of view at the bottom surface of arteries at an acquisition rate of 3 Hz using Fluoview software (version 3.5). Following a baseline of > 30 s, 0.1–100 nM bradykinin was added to generate cumulative CRCs in a static bath. Peak responses were observed within 90 s. Up to 6 cells per field of view were analyzed and averaged to give one *n* value per artery. Data were analyzed offline using MetaMorph software (version 7.7.4.0, Molecular Devices). Subcellular regions of interest (diameter ~5 μm) were positioned both within active cells and away from cells (latter for background intensity) to obtain fluorescence intensity over time. Background-subtracted raw data are expressed as relative fluorescence (F/F_0_) by dividing fluorescence intensity (F) by an average baseline fluorescence intensity F_0_. Values are summarized as the mean ± SEM, with *n* representing the number of arteries studied.

#### SMC intracellular Ca^2+^ studies

Animal use was approved by the University of Oxford Ethical Committee and complied with the Animals (Scientific Procedures) Act 1986. Animals were housed in a temperature-controlled environment with a 24-h light-dark cycle and water *ad libitum*. These studies comply with ARRIVE guidelines (24, 25). Male Wistar rats (230–280 g) were killed by exposure to rising concentration of CO_2_, and confirmed by cervical dislocation [as specified by Schedule 1 of the Animals (Scientific Procedures) Act 1986, UK]. The heart was pinned and incision into the right ventricle exposed the septal artery, which was dissected and mounted into a confocal wire myograph (model 120CW, Danish Myo Technology A/S) and in Krebs solution containing (in mM): 118 NaCl, 25 NaHCO_3_, 3.6 KCl, 1.2 MgSO_4_·7H_2_O, 1.2 KH_2_PO_4_, 1.25 CaCl_2_, 11 glucose and gassed with 21% O_2_, 5% CO_2_, with N_2_. The solution temperature was raised to 37°C, and the artery normalized to a resting tension equivalent to that generated at 90% of the diameter of the vessel at 80 mmHg (26). Following an equilibration period of 1 h, endothelial function was assessed by >90% relaxation to 100 nM acetylcholine from pre-constriction with phenylephrine. Viable arteries were loaded with the calcium-sensitive fluorescent dye Calbryte 520 (AAT Bioquest) [2.5 μM; dissolved in DMSO and 0.03% (w/v) Pluronic F-127] for 30 min at 30°C, then incubated in Krebs buffer for 30 min at 37°C to allow de-esterification. After excitation at 488 nm, the fluorescence emission intensity at 513–563 nm was recorded from the bottom surface of arteries using a spinning disc confocal microscope (Yokogawa CSU22) fitted with an Andor iXON DV887ECS-BV camera mounted on an Olympus IX70 inverted microscope using a water immersion objective (x40, aperture 0.8, working distance 3.3 mm; Olympus) and images (430 x 420 pixels, 35 Hz) were stored for offline analysis (iQ version 3.5, Andor Bioimaging Division, UK; MetaMorph version 7.7.4.0, Molecular Devices). Following background subtraction, average relative changes in [Ca^2+^] were calculated as changes in intensity of fluorescence divided by fluorescence at time 0 s (F/F_0_), within selected cell regions and the whole field. Data are expressed either as relative fluorescence (F/F_0_) or the frequency of Ca^2+^ events observed per min.

#### Electrophysiology in *ex vivo* tensioned coronary IMCAs

Porcine RA-IMCAs were dissected as for pressure myography, but were instead mounted in a wire myograph, for simultaneous measurement of SMC membrane potential and isometric tension, as previously for rat coronary arteries (26). Arteries (~2 mm long) were normalized to a resting tension equivalent to that generated at 90% of the inner diameter of the vessel at 80 mmHg, treated with 45 mM K^+^ and washed. SMC membrane potential and tension were recorded through a pre-amplifier (Neurolog system, Digitimer Ltd., U.K.) linked to a MacLab data acquisition system (AD Instruments Model 4e, New Zealand) and LabChart software (v7.2.5, AD Instruments, New Zealand). Individual SMCs were impaled with sharp glass microelectrodes (backfilled with 2 M KCl; tip resistances *circa* 60 MΩ), observed as a rapid deflection toward the resting membrane potential, near −50 mV. The chamber was maintained at 37°C.

### 3D structure of *ex vivo* pressurized coronary IMCAs

#### Electron microscopy

IMCAs processed for electron microscopy were fixed at the end of functional assessment for myogenic tone and vasomotor responses, including conducted dilation, as previously described (18). In this study the previously obtained serial SBF-SEM images acquired were handled and processed for segmentation with Microscopy Image Browser (MIB), Helsinki (27). Individual cells were followed in all three dimensions for reconstructions with MIB. The cells were manually traced in MIB using the “brush” drawing tool and aided by interpolation. The individual cell models were compiled using Imaris (version 9.8.0) for presentation.

#### Confocal fluorescence microscopy

Studies of K_Ca_ channel expression were carried out following fixation of cannulated IMCAs at 80 mmHg with 2% (w/v) paraformaldehyde for 10 min at 36.6 ± 0.3°C, washing with phosphate-buffered saline before further study. Whilst still cannulated, IMCAs were exposed to antibodies in the incubation chamber to label from the outside of arteries, and were also pumped into the lumen of arteries to label the ECs and inner SMC layers, as previously (18, 28). Primary antibodies were as follows: 1:100 rabbit polyclonal anti-rat K_Ca_3.1 (aa 350–363; Alomone Laboratories, APC-064); 1:100 mouse monoclonal anti-human K_Ca_3.1 (third extracellular loop; Alomone Laboratories, ALM-051); and 1:100 rabbit polyclonal anti-human K_Ca_2.3 (aa 2–21, Alomone Laboratories, APC-025). Secondary antibodies were as follows: 1:100 goat anti-rabbit IgG, Invitrogen, A-11008; or 1:100 chicken anti-mouse IgG, Invitrogen, A-21200. Nuclei and elastin (including the internal elastic lamina, IEL) were stained with 15 μM propidium iodide and 200 nM Alexa Fluor 633 hydrazide (Molecular Probes, A-30634), respectively (28). Arteries were excited at 488, 546, and 633 nm; the fluorescence emitted at 505–525, 560–620, and 655–755 nm was acquired through a water immersion objective (1.15 NA, Olympus, 1,024 × 1,024 pixels) using a laser scanning confocal microscope (FV1000 or FV1200; Olympus). z-stacks through the artery wall were obtained at 0.20-μm increments by using Fluoview Software (FV10-ASW 3.0; Olympus) and reconstructed in Imaris Software (version 8.0.2; Bitplane).

### Statistical analysis

Statistical analysis was performed using GraphPad Prism software (version8, GraphPad Software, La Jolla, USA), where *P* < 0.05 was considered significant. All specimens collected were analyzed and no experimental data that passed the inclusion criteria (≥ 10 % myogenic tone and dilation to bradykinin) were excluded from the study. Formal statistical comparisons on paired data first tested for Gaussian distributions (D'Agostino and Pearson omnibus normality test), which confirmed non-parametric tests should be used. Values are given as mean ± SEM unless otherwise specified.

## Results

### Characteristics of human and porcine RA-IMCAs

The human and porcine RA appendage biopsies used for experiments in this study were a subset of those used previously (18) (Table 1) with 2 additional patients (Table 2, data not included in diameter summaries). The maximum inner diameter of human arteries was 153 ± 6 μm and they developed 23.3 ± 1.8% myogenic tone to 117 ± 5 μm (*n* = 45). For comparison purposes, a limited number of IMCAs were also obtained from human organ donors (OD-RA-IMCAs). These had a maximum inner diameter of 152 ± 10 μm and each developed >10% MT (32.1 ± 7.2% MT, *n* = 3) (18). Of the 39 porcine biopsies, all arteries developed >10% MT and fully dilated to bradykinin, matching the criteria for inclusion. The maximum inner diameter of porcine arteries was 203 ± 11 μm developing 25.4 ± 1.5% MT to reach stable resting diameters of 154 ± 10 μm (*n* = 39).

### Characteristics of human and porcine LV-IMCAs

We were able to source a limited number of ventricular biopsies obtained from organ donors, and pigs at the time of sacrifice, for comparison of bradykinin responses between heart chambers and across species. The left ventricular IMCAs from human organ donors (OD-LV-IMCAs) had a maximum inner diameter of 272 ± 56 μm and each developed >10% MT (26.7 ± 0.9% MT, *n* = 4) (18). The porcine LV-IMCAs had a maximum inner diameter of 248 ± 38 μm and each developed >10% MT (22.9 ± 3.6% MT, *n* = 3) (18).

### Comparison of EC-dependent dilation in human and porcine RA/LV-IMCAs

**Figure 1:**
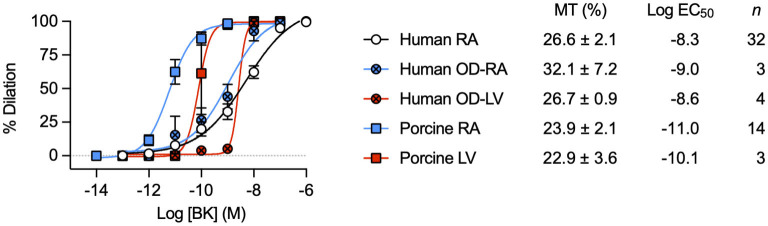
Characterization of dilation to bradykinin in IMCAs mounted in a pressure myograph. Comparison of control concentration-dependent BK vasodilation responses across species and heart chambers. Data from human and porcine RA-IMCAs are compared to organ donor (OD) RA and left ventricle (LV) arteries, and porcine LV arteries. Values specific to the experiments performed in this study for both MT and the non-organ donor RA-IMCA concentration response curves to BK are modified from Dora et al. (18).

The percentage concentration-dependent vasodilation of developed MT to bradykinin was remarkably similar between RA samples from patients undergoing elective cardiac surgery and from the organ donors (Figure 1). The mean age of each group was similar (elective surgery 62 ± 12 years, *n* = 45; organ donors 63 ± 8 years, *n* = 3; mean ± SD). These data support the use of patients undergoing this type of surgery (Table 1), as individuals without significant coronary microvascular disease. The magnitude of developed MT in porcine RA-IMCAs was similar, yet the concentration-dependent vasodilation to bradykinin in human arteries was *circa* 50-fold (LV) to 500-fold (RA) right-shifted compared to the porcine arteries (Figure 1). The similarity in MT and EC-dependent characteristics of organ donor LV-IMCAs, organ donor RA-IMCAs and elective surgery patient RA-IMCAs gave security to proceed with the more readily available elective surgery patients for the remainder of this study.

### Structural observations in coronary IMCAs

**Figure 2:**
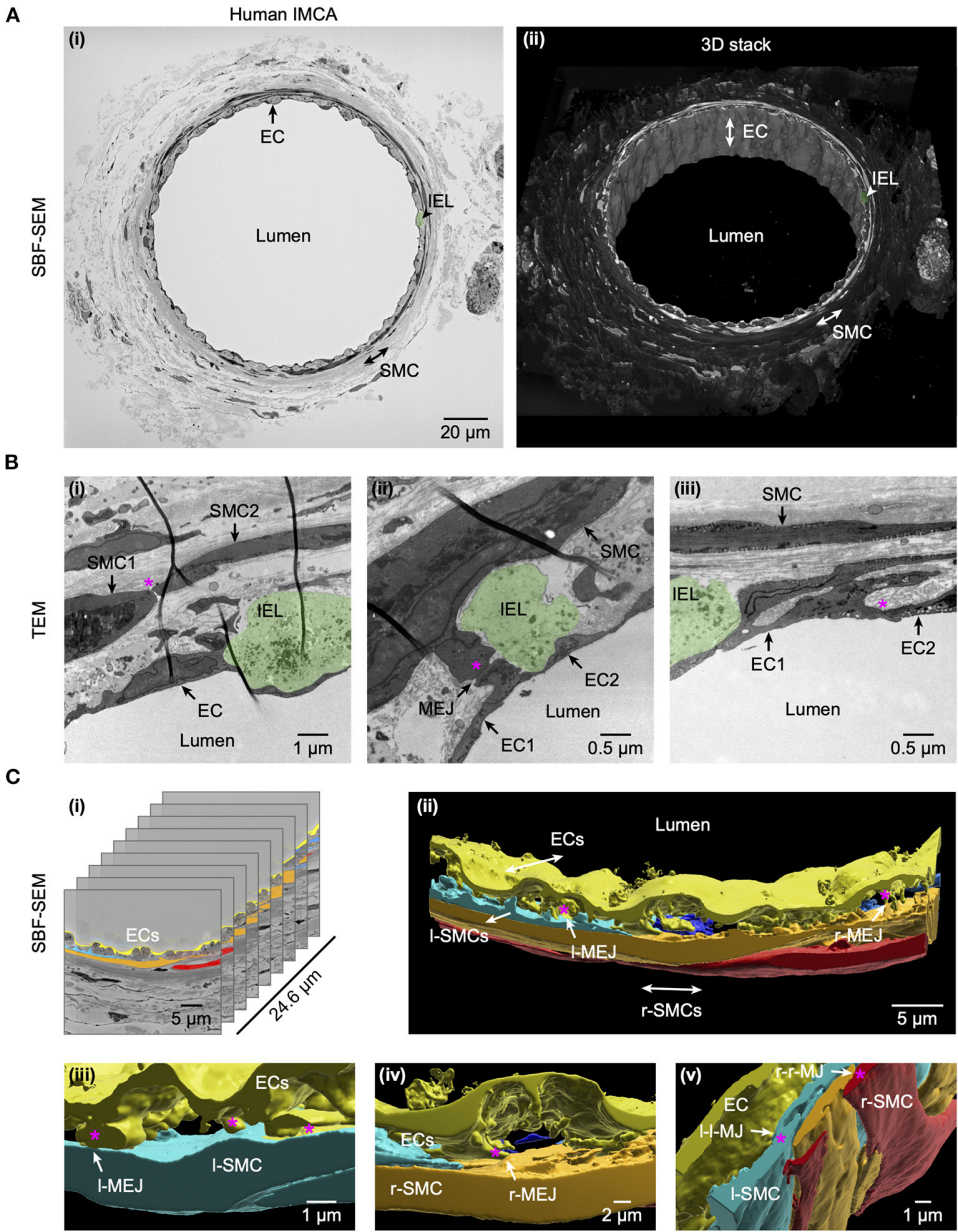
Cell-cell contact sites in a human RA-IMCA with good contractile function. The artery (Artery #1, Table 2) was first used for functional experiments (included in Figures 3, 7) and then while still cannulated and pressurized, processed for both serial block face scanning electron microscopy (SBF-SEM, **A**) and transmission electron microscopy (TEM, **B**). At low resolution **(A)** it is clear that 1–2 layers of SMCs surround the ECs and internal elastic lamina (IEL). Arrows indicate the same EC, and position of SMCs and IEL in both the 2D- (left) and 3D- (right) images. The IEL comprises longitudinal strings of elastin (examples in Figures 5A, 6A,B), one example is highlighted in green in all panels. A movie through the 3D z-stack at low and higher resolution is available at Dora et al. (18). Higher resolution images **(B)** were obtained before processing for SEM, and showed clear contact sites between SMCs, between SMC and ECs, and between ECs, an example indicated by the asterisk in each panel. Representative of 5 arteries which developed myogenic tone and responded to BK (18). **(C)** The cell-cell contact sites were also visible in the SBF-SEM images (Supplementary Video 1). Individual cells were 3D rendered and are shown superimposed on the original images **(i)** and as the reconstructed artery walls **(ii–v)**. Longitudinally arranged SMCs (l-SMCs) are shown in blue (see Figure 12), and circumferential radial SMCs (r-SMCs) in orange and red; each of which contacted each other and endothelial cells. Examples are indicated by r-MEJ, r-SMC to EC junction; l-MEJ, l-SMC to EC junction; l-l-MJ, l-SMC to l-SMC junction; r-r-MJ, r-SMC to r-SMC junction.

Some of the arteries used to assess conducted vasodilation to bradykinin were subsequently fixed and examined microscopically at high resolution. Cell-cell contacts were clearly visible between smooth muscle cells, endothelial cells and between the two cell types (Figure 2). These sites of contact are the potential location of gap junctions, which are essential for local and conducted homo- and heterocellular electrical coupling and hence underpin conducted vasodilation.

### Conducted vasodilation initiated by focal application of bradykinin

**Figure 3:**
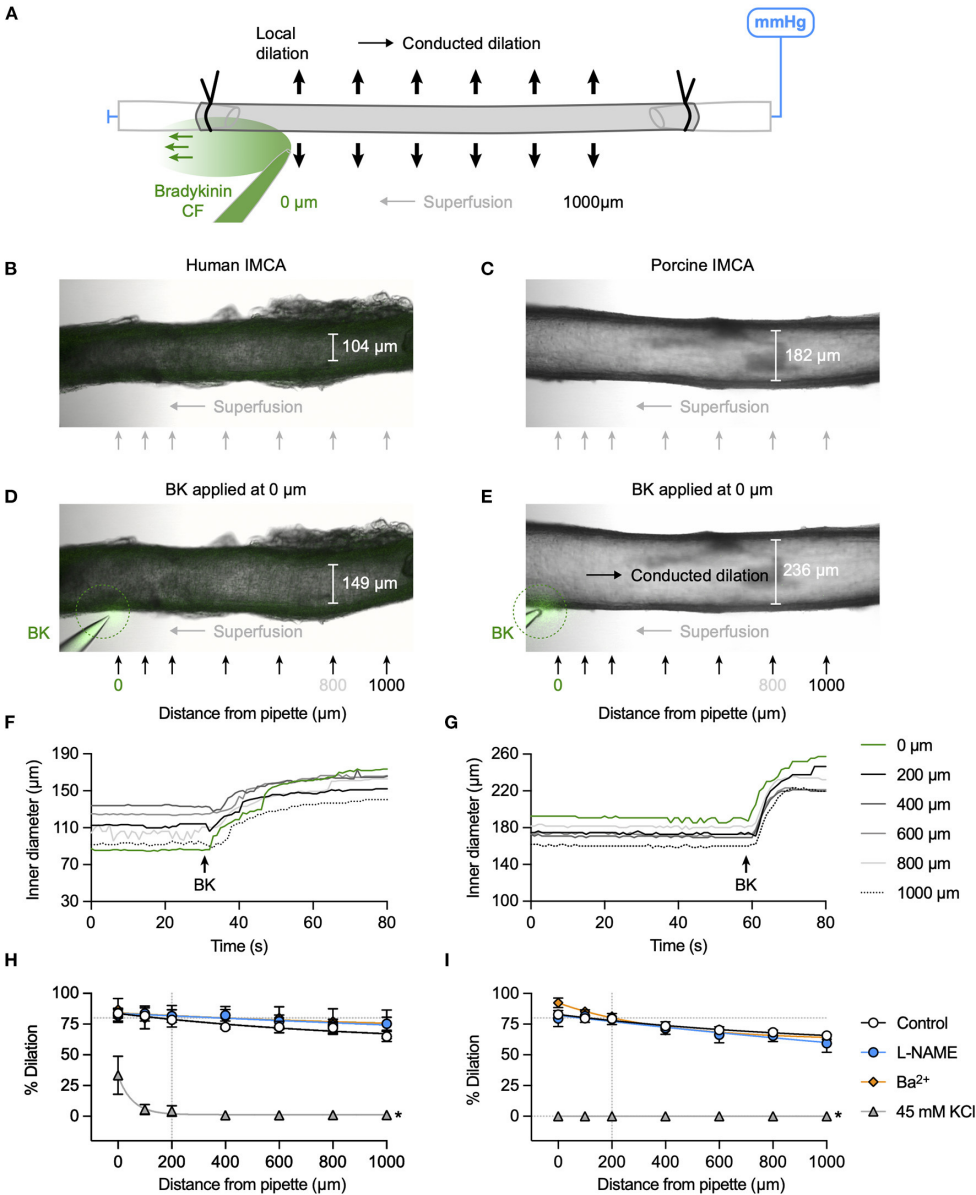
K^+^ channel-mediated vasodilation underlies conducted dilation of human and porcine RA-IMCAs. **(A)** Schematic of experimental setup with direction of flow of bradykinin (BK) indicated by carboxyfluorescein (CF). Micrographs showing an isolated, cannulated and pressurized human **(B**) and porcine **(C)** artery. Focal application of BK (and CF, green) to the downstream end of the artery against the direction of superfusion flow caused local and conducted dilation in both human **(D)** and porcine **(E)** arteries. The corresponding time course of responses are shown in **(F,G)**, and Supplementary Videos S2, S3. A bolus of bradykinin was delivered at the point indicated by the arrow, and simultaneous inner diameter measured locally (0 μm) and up to 1,000 μm upstream, positions indicated by arrows in **(D,E)**. The same human artery used for K_Ca_3.1 immunolabel in Figure 6A. Summary graphs show that compared to control (*n* = 9, 14) neither L-NAME (100 μM, *n* = 3, 6) nor Ba^2+^ (30 μM, *n* = 3, 3) affected local or conducted dilation, whereas depolarization to 45 mM KCl abolished conducted dilation (*n* = 3, 6) in human **(H)** and porcine **(I)** arteries, respectively. Non-parametric mixed effects analysis with Sidak's multiple comparison test; *P < 0.05 vs. control.

**Figure 4:**
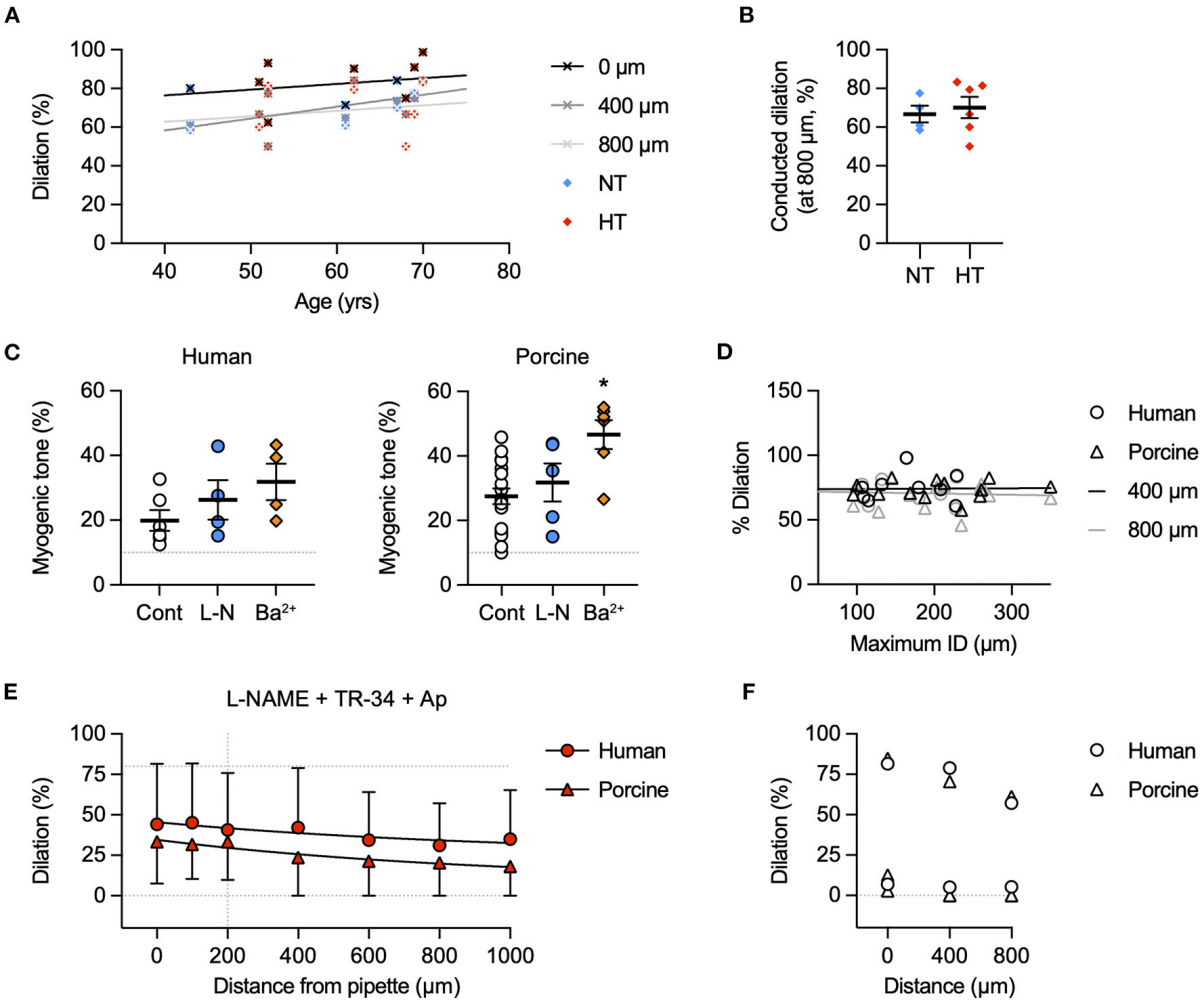
Possible factors influencing conducted dilation. **(A)** Effect of patient age on conducted dilation to bradykinin in RA-IMCAs. Paired control data from Figure 3 for each patient are aligned with patient age. Simultaneous values for dilation at 0, 400, and 800 μm are shown. There was no significant correlation with age in this cohort of 10 patients, each undergoing valve surgery. **(B)** Patients were separated according to whether they were being treated for hypertension (HT) or were normotensive (NT). There was no difference in conducted dilation at 800 μm. Values are shown at the time point where the response at 200 μm was 80% of maximum diameter (to normalize responses between patients). **(C)** Effect of inhibitors on baseline myogenic tone in human and porcine RA-IMCAs. Non-parametric mixed effects analysis with Dunn's multiple comparison test; *, *P* < 0.05 vs. control. **(D)** Effect of RA-IMCA maximum inner diameter (ID) on the conducted dilation to bradykinin observed at 400 μm and 800 μm upstream from the delivery pipette. Paired control data from Figure 3 for each patient are aligned with maximum ID. **(E)** Effect of the presence of the K_Ca_ channel inhibitors TRAM-34 and apamin (in addition to L-NAME) on local and conducted vasodilation to bradykinin in human (*n* = 2) and porcine (*n* = 3) RA-IMCAs. **(F)** The data sets could be separated to those where the K_Ca_ channel inhibitors had a marked effect (3 of 5 arteries) and those where there was little effect. The individual data from **(E)** for responses at 0 μm (local), 400 μm and 800 μm (conducted) are shown.

The focal delivery of bradykinin (indicated by co-release of fluorescent carboxyfluorescein) to one end of either human or porcine RA-IMCAs evoked vasodilation that spread along the arteries with minimal decline for at least 1,000 μm from the site of application (Figure 3). The age of patients did not influence the decay with distance along the artery (*n* = 9, Figure 4A). The local and conducted vasodilation to bradykinin was not affected by the presence of 100 μM L-NAME to block endothelial NO synthase or 30 μM Ba^2+^, a concentration that selectively blocks vascular inwardly rectifying K channels (K_IR_). In contrast, vasodilation to bradykinin was abolished by raised extracellular K^+^ (45 mM). These experiments are summarized in Figures 3H,I. Experiments were also performed in the presence of TRAM-34 and apamin to block endothelial K_Ca_ channels. As shown previously in human RA-IMCAs (10), both local and conducted vasodilation were markedly reduced (Figure 4E). We did not pursue this aspect further, but it was clear there was some variability as in combination TRAM-34 (to block IK_Ca_, K_Ca_3.1) and apamin (to block SK_Ca_, K_Ca_2.3) either blocked local and conducted responses (3 of 5 arteries) or had little influence (Figure 4F).

### Bradykinin activates EC Ca^2+^ elevation, K_**Ca**_ channels, and SMC hyperpolarization in human and porcine RA-IMCAs

**Figure 5:**
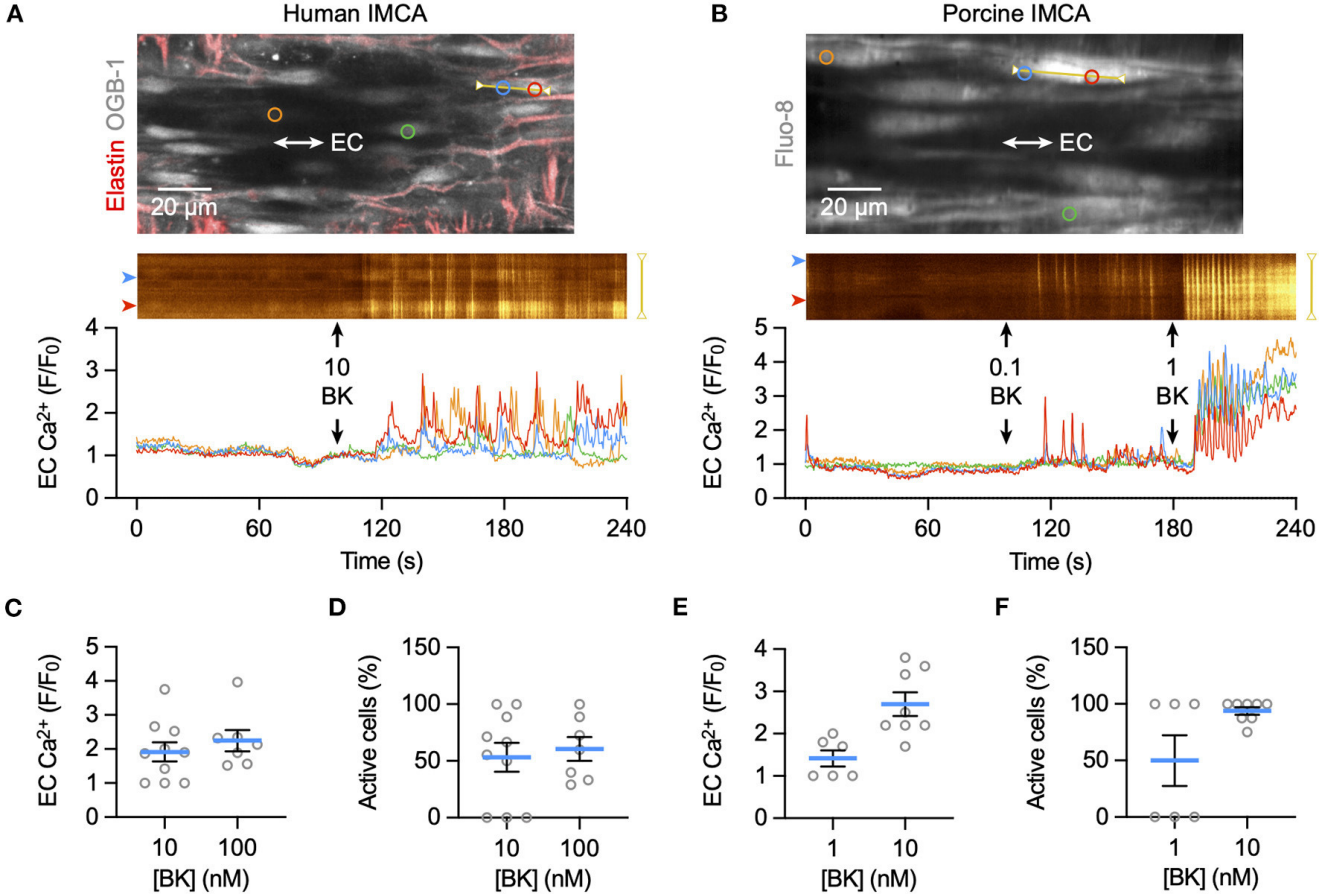
EC Ca^2+^ increase to bradykinin in *ex vivo* human and porcine RA-IMCAs. Confocal micrographs showing an isolated, cannulated and pressurized human IMCA **(A)** and porcine IMCA **(B)** loaded with Ca^2+^ indicator to image ECs near the bottom plane of the artery. Longitudinal (IEL) and radial strands of elastin are visible in the human artery (labeled with AF-633, red). The spatio-temporal characteristics of EC Ca^2+^ events are shown in line-scan analysis of each yellow line, and the average fluorescence intensity within each colored circle as F/F_0_ in the traces below. The addition of each concentration (nM) of bradykinin (BK) to the chamber is indicated by arrows. Summary of EC Ca^2+^ responses to BK as F/F_0_ and percentage of active cells, in human **(C,D)** and porcine **(E,F)** arteries.

**Figure 6:**
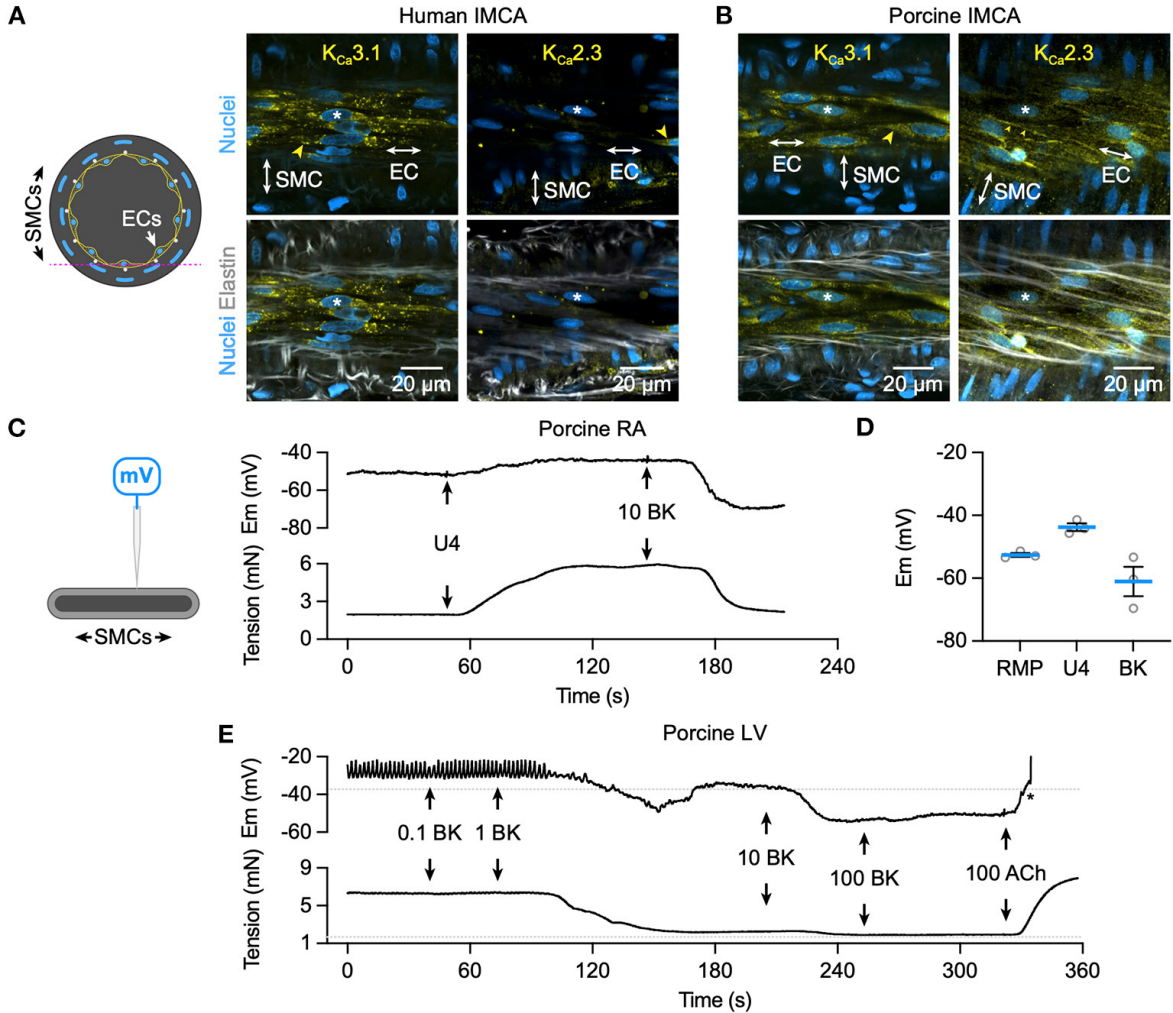
K^+^ channel expression and hyperpolarization to bradykinin in human and porcine IMCAs. Confocal micrographs of immunolabelling for K_Ca_3.1 and K_Ca_2.3 in isolated, cannulated and pressurized human **(A)** and porcine **(B)** RA-IMCAs with myogenic tone and full dilation to BK. Punctate and diffuse K_Ca_3.1 label was evident in the ECs of human and porcine arteries, whereas K_Ca_2.3 was less clear in human IMCAs, and highly expressed at EC borders of porcine IMCAs (yellow arrowheads). The elastin was dense in human arteries, with the internal elastic lamina (IEL) seen as longitudinal strings in the porcine arteries. Representative of at least 3 arteries for each label; asterisks indicate corresponding nuclei in upper and lower panels. The pink dashed line in the schematic represents the focal plane. **(C)** The schematic indicates a sharp microelectrode impaled into a SMC of a porcine RA-IMCA mounted for isometric tension recording. Under control conditions the thromboxane mimetic U46619 (0.6 μM) depolarized and contracted arteries, and BK (10 nM) caused hyperpolarization and relaxation **(C)**, summarized in **(D)**. **(E)** Addition of L-NAME depolarized and contracted porcine left ventricular (LV)-IMCAs. Under these conditions BK (0.1 nM to 100 nM) repolarized and relaxed the artery. Addition of 100 nM acetylcholine (ACh) depolarized and contracted the artery. The asterisk indicates when the electrode came out of the cell. The RMP and tension prior to the addition of L-NAME (100 μM) are indicated by dashed lines. Drugs were added to a static bath at the arrows. RMP, resting membrane potential.

Bradykinin increased cytoplasmic Ca^2+^ in endothelial cells of myogenically constricting, pressurized RA-IMCAs. Human IMCAs were less sensitive than porcine arteries, with a threshold around 10 nM to initiate Ca^2+^ waves in *circa* 50% of cells, which appeared to be a maximum effect as no further increase followed 100 nM (Figures 5A,C,D). In contrast, in porcine IMCAs, 0.1 nM bradykinin initiated Ca^2+^ events in 50% of cells, rising to include all cells with 1 nM bradykinin (Figures 5B,E,F). The ability of low nM bradykinin to evoke EC Ca^2+^ events correlated with the greater sensitivity of vasodilation observed in porcine compared to human arteries (Figure 1). ECs in both species showed discrete immunolabelling for both K_Ca_3.1 and K_Ca_2.3, respectively the intermediate- and small-conductance K_Ca_ channels responsible for endothelium-dependent hyperpolarization (EDH; Figures 6A,B). The expression of K_Ca_3.1 was consistent and abundant in ECs of both species. In contrast, the expression of K_Ca_2.3 although abundant in porcine ECs was less evident in ECs of human IMCAs, with some punctate label detected in the SMCs. Bradykinin has been shown previously to stimulate SMC hyperpolarization in human RA-IMCAs pre-constricted with endothelin-1 (12). Here, porcine RA-IMCAs pre-constricted with the thromboxane mimetic U46619 (0.6 μM) under isometric conditions were exposed to 10 nM bradykinin, which stimulated 17.3 ± 4.1 mV hyperpolarization (initial resting potential −52.6 ± 0.6 mV, with U4: −43.7 ± 1.2 mV, *n* = 3; Figures 6C,D), and completely reversed U46619 pre-constriction. Bradykinin also hyperpolarized a porcine RA-IMCA with myogenic tone, 10 nM bradykinin fully reversing tone and maximally hyperpolarizing the artery (Figure 6E).

### Vasodilation to bradykinin is mediated by EDH not NO in human and porcine RA-IMCAs

**Figure 7:**
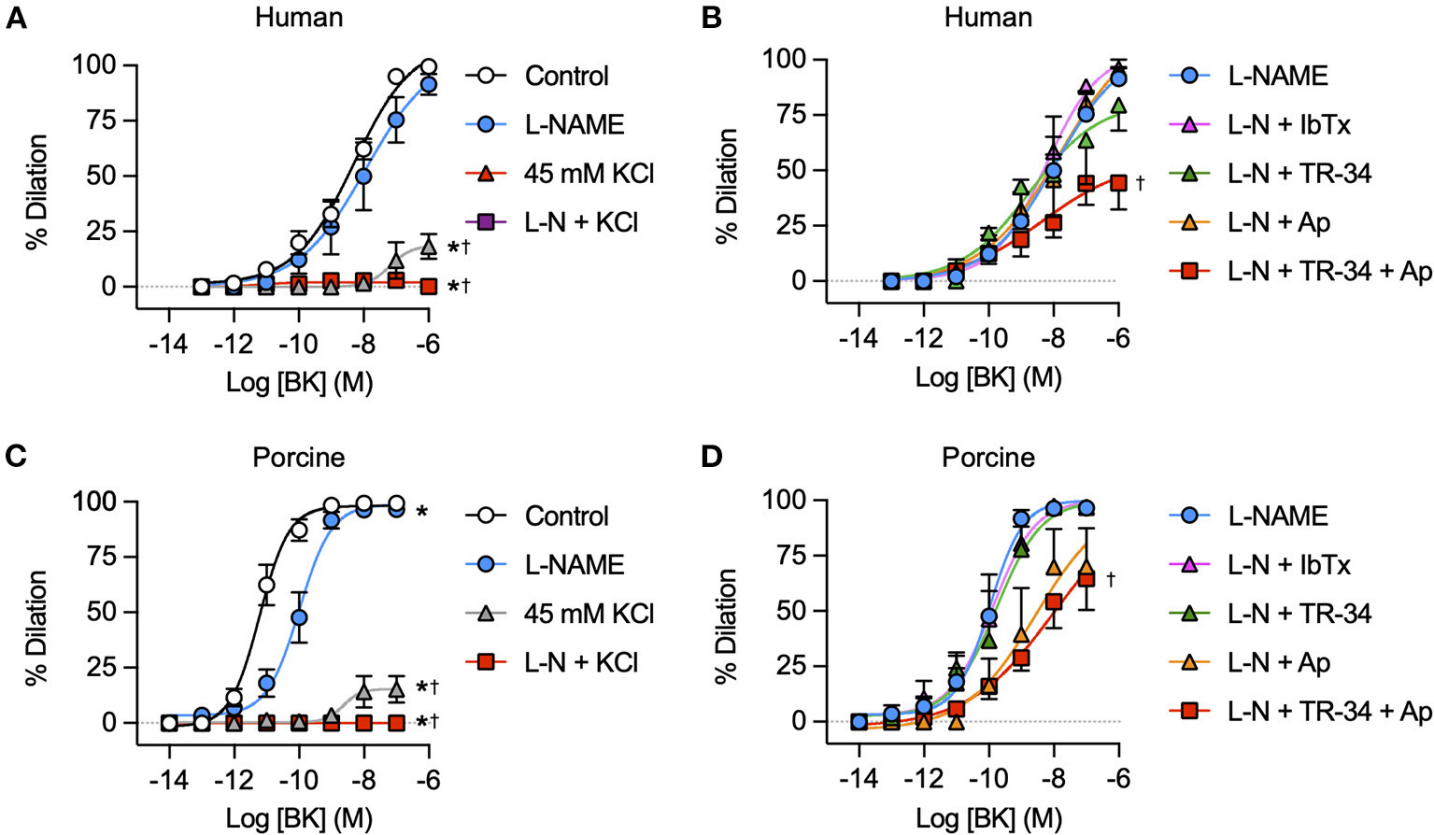
K^+^ channel mediated vasodilation of human and porcine RA-IMCAs. Concentration response curves to BK in the presence and absence of NOS inhibitor L-NAME (L-N, 100 μM) and/or 45 mM isotonic KCl and selective K_Ca_ channel inhibitors iberiotoxin (IbTx, BK_Ca_, 0.1 μM), TRAM-34 (TR-34, IK_Ca_, 1 μM) and/or apamin (Ap, SK_Ca_, 0.1 μM) in human **(A,B)** and porcine **(C,D)** RA-IMCAs (*n* = 3-32). Non-parametric mixed effects analysis with Sidak's multiple comparison test; *P < 0.05 vs. control;^†^*P* < 0.05 vs. L-NAME.

Block of NO synthase with L-NAME did not modify bradykinin-mediated vasodilation in either human (Figure 7A) or porcine IMCAs (Figure 7C), whereas raising extracellular K^+^ to block hyperpolarization effectively abolished responses to bradykinin. In the presence of L-NAME, the K_Ca_ subtype activated by bradykinin was probed with specific blocking agents. Vasodilation was significantly reduced in both species by exposure to a combination of TRAM-34 and apamin consistent with the known role of these channels in mediating EDH across a wide range of species. Iberiotoxin, which acts specifically to block SMC BK_Ca_ channels was without effect (Figures 7B,D).

### Vasodilation to adenosine is mediated by hyperpolarization in porcine RA-IMCAs

**Figure 8:**
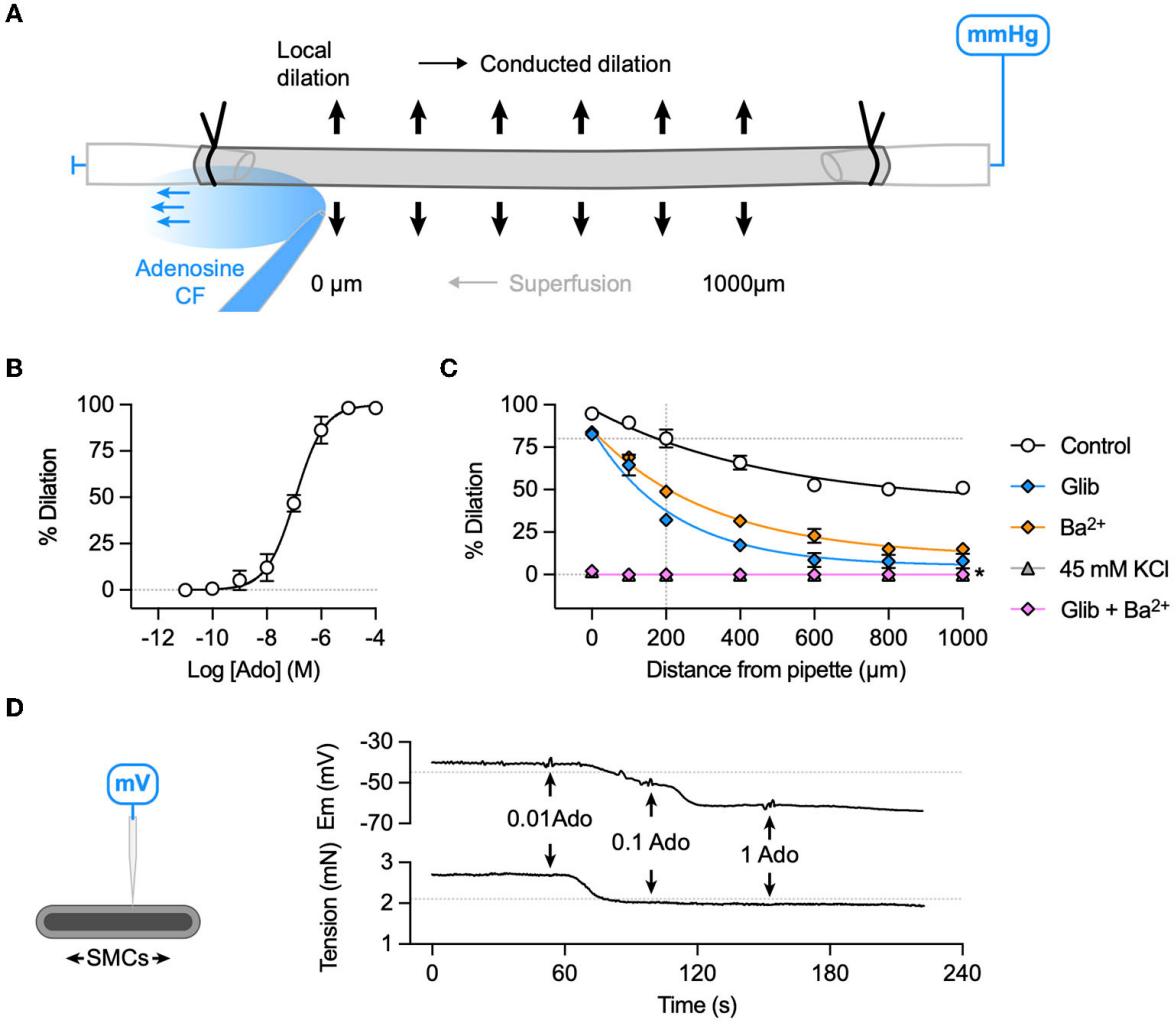
K^+^ channel-mediated vasodilation underlies conducted dilation to adenosine in porcine RA-IMCAs. **(A)** Schematic of experimental setup with direction of flow of adenosine (Ado) indicated by carboxyfluorescein (CF). **(B)** Concentration-dependent dilation to adenosine added to a static bath (log EC_50_:−6.9; *n* = 3). **(C)** A bolus of adenosine was delivered at the point indicated by the arrow, and simultaneous inner diameter measured locally (0 μm) and up to 1,000 μm upstream. Summary graphs show that compared to control (*n* = 5) either glibenclamide (Glib, 5 μM, *n* = 5) or Ba^2+^ (30 μM, *n* = 3) alone reduced conducted dilation, whereas the two together or depolarization to 45 mM KCl abolished conducted dilation (*n* = 3, 3), respectively. For control data, simultaneous diameter measurements were made at the time when adenosine evoked 80% of maximal dilation at 200 μm upstream from the pipette and visible CF (indicated by intersection of dashed lines). Non-parametric mixed effects analysis with Sidak's multiple comparison test; *P < 0.05 vs. control. **(D)** The schematic indicates a sharp microelectrode impaled into a SMC of a porcine RA-IMCA mounted for isometric tension recording. In the presence of L-NAME, adenosine (0.01 to 1 μM, *n* = 1) hyperpolarized and relaxed the artery. The resting membrane potential (−45.0 mV) and tension (2.1 mN) prior to the addition of L-NAME (100 μM) are indicated by dashed lines.

For comparison to bradykinin, the partially endothelium-dependent coronary vasodilator adenosine (29) was used to study local and conducted vasodilation in porcine RA-IMCAs. The experimental approach was the same as for bradykinin (Figure 8A), and concentration-dependent vasodilation was observed (Figure 8B), with EC_50_ ~0.1 μM. Conducted vasodilation to adenosine was robust in the porcine RA-IMCAs and blocked in the combined presence of Ba^2+^ and glibenclamide (to inhibits K_*ATP*_ channels). Individually, these agents only partially inhibited vasodilation, while raised extracellular K^+^ blocked local and conducted dilation (Figure 8C). Adenosine stimulated concentration-dependent hyperpolarization, which was associated with relaxation (Figure 8D).

### l-SMCs and r-SMCs are coupled

**Figure 9:**
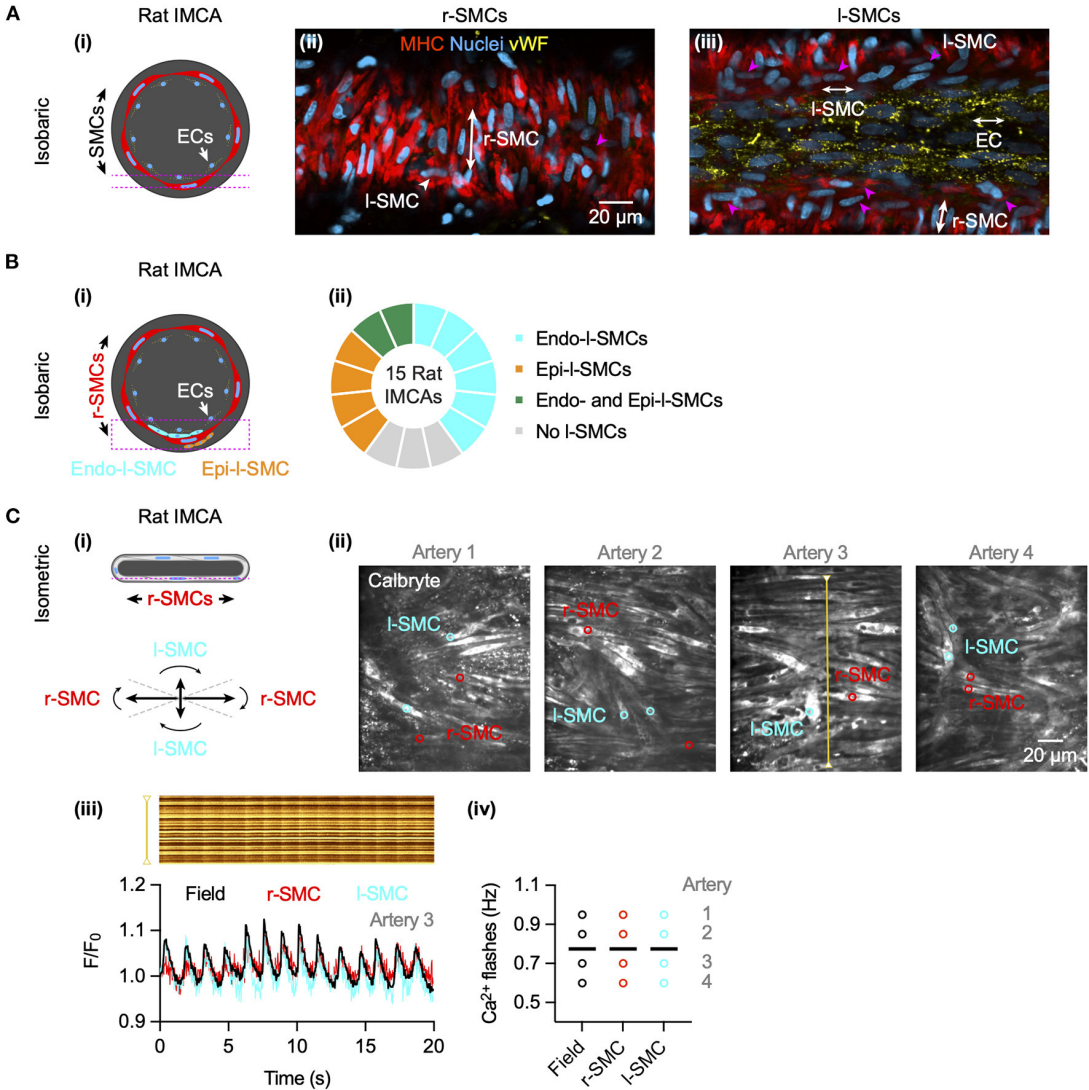
Cell-cell contacts facilitate electrical communication in coronary arteries. **(A)** Rat septal IMCAs each developed myogenic tone when mounted for pressure myography (25.1 ± 2.8%, *n* = 15). Arteries were fixed and immunolabelled for SM-myosin heavy chain II to label SMCs and von Willebrand factor (vWF) for ECs. Both r-SMC and l-SMCs were visible, the latter clearly separate from ECs. The pink dashed lines in the schematic **(i)** show the focal planes for imaging the SMCs **(ii)** and ECs **(iii)** of the same artery. Pink arrowheads show examples of l-SMCs. Representative of 12 arteries (of 15 imaged) with l-SMCs. **(B)** In pressurized arteries **(i)**, l-SMCs were observed between the ECs and r-SMCs (endo-l-SMCs) and outside the r-SMCs (epi-l-SMCs). **(ii)** Most arteries had endo-l-SMCs (12 of 15), and some had both endo-l-SMCs and epi-l-SMCs (2 of 15). 3 arteries had <5 l-SMCs in the z-stack (region indicated by dashed pink box in **(i)**, considered no l-SMCs. **(C)** In arteries set up in a confocal wire myography **(i)** SMCs were imaged at the bottom surface (focal plane indicated by dashed pink line). The r-SMCs were often in the same focal plane as the l-SMCs (angles for categorization shown). **(ii)** Confocal micrographs of 4 arteries are shown with regions used for average fluorescence (indicated by colored circles). **(iii)** The spatio-temporal characteristics of spontaneous SMC Ca^2+^ flashes in Artery 3 are shown in line-scan analysis of the yellow line (upper), and corresponding average fluorescence intensity within a r-SMC (red) and l-SMC (cyan) and the whole field (black) as F/F_0_ below. Summary data are shown in **(iv)**, each individual artery had simultaneous Ca^2+^ flashes within all r-SMCs and l-SMCs, the frequency only varying between arteries (mean indicated by bars; 0.78 ± 0.08 Hz, *n* = 4).

The l-SMCs in the wall of human and porcine RA-IMCAs (Figure 2) (18) have not been fully characterized in terms of their physiological role. Their orientation and intercellular homo- and heterocellular contacts lends them to facilitating the passage of current through the wall of coronary IMCAs. We have previously shown that SMCs in rat septal intramuscular (coronary) arteries exhibit T-type and L-type VGCC-dependent transient depolarizing spikes which are driven once a threshold depolarization is surpassed (26). The presence of l-SMCs was confirmed by immunolabel (Figures 9A,B) and the coupling between r-SMCs and l-SMCs was demonstrated as synchronous Ca^2+^ flashes in both cell types (Figure 9C).

### Structural observations in coronary micro-arteries with contractile dysfunction

**Figure 10:**
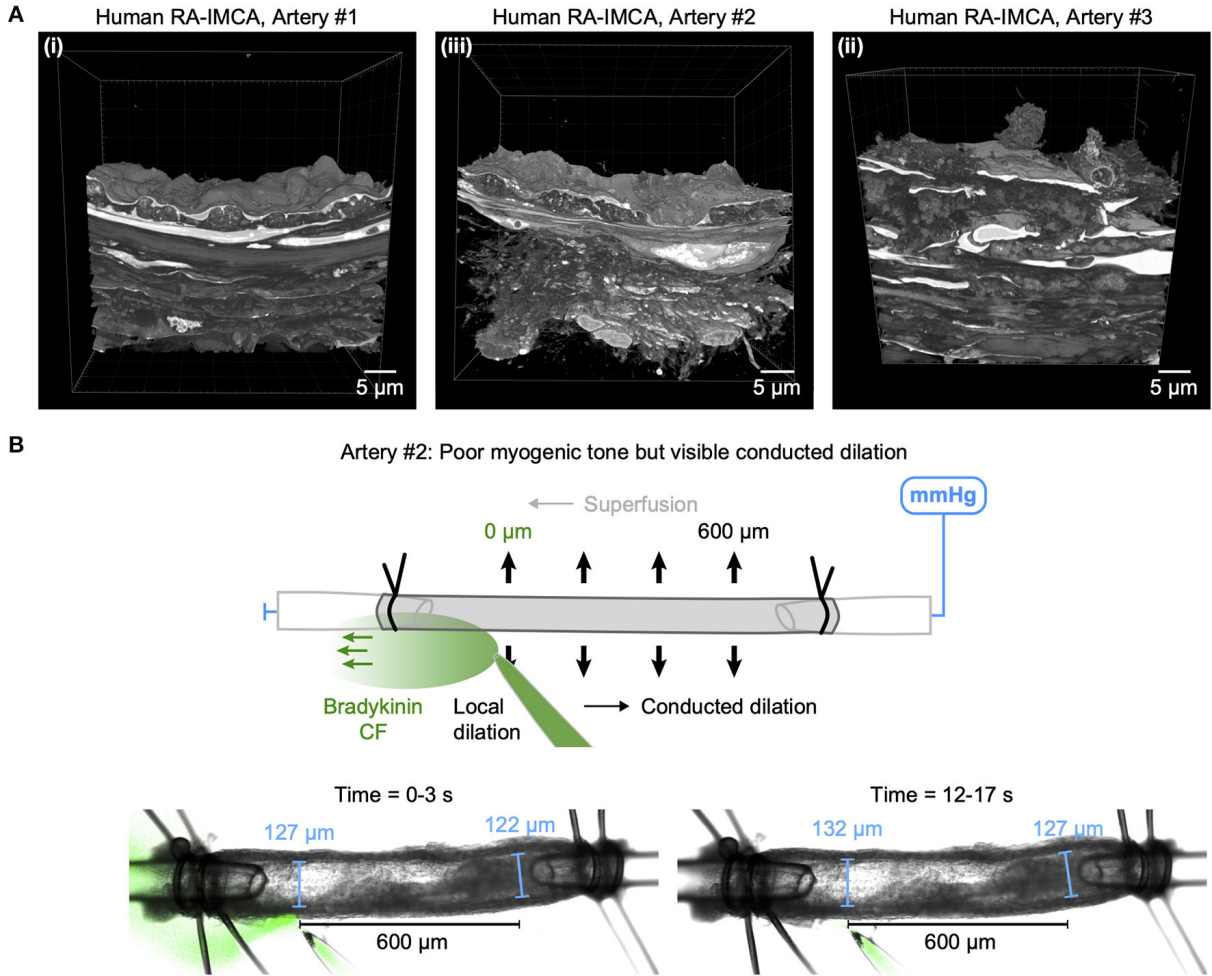
Cell-cell contacts enable conducted dilation in a human RA-IMCA with poor contractile function. **(A)** SBF-SEM 3D stacks of the wall of three RA-IMCAs, Artery #1 to #3 (Table 2), also shown in in Dora et al. (18), including z-stack movies. **(B)** While Artery #2 did not develop good myogenic tone (<10%), it was nevertheless used to assess bradykinin-mediated conducted dilation, which was clearly visible at 600 μm upstream from the pipette.

**Figure 11:**
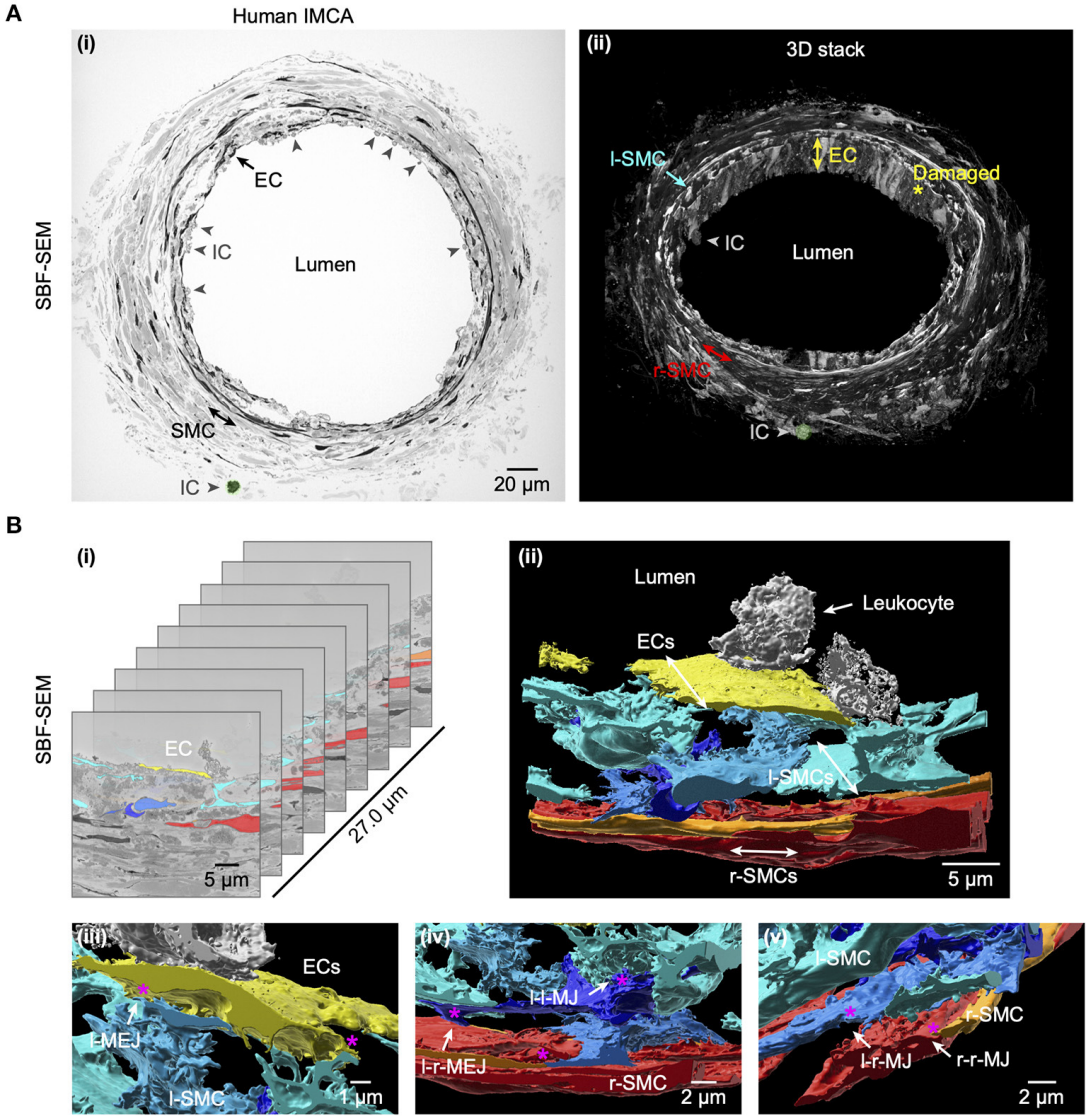
Cell-cell contact sites in a human RA-IMCA with contractile dysfunction. The artery (Artery #3, Table 2) did not develop myogenic tone but was processed for serial block face scanning electron microscopy. At low resolution **(A)** it is clear that many ECs are damaged and inflammatory cells (ICs) have adhered to the artery wall, the same IC is highlighted in green in both the 2D- (left) and 3D- (right) images. A movie through the 3D z-stack at low and higher resolution is available at Dora et al. (18). **(B)** The cell-cell contact sites were visible in the SBF-SEM images. Individual cells were 3D rendered and are shown superimposed on the original images **(i)** and as the reconstructed artery walls **(ii–v**; Supplementary Video 4). Multiple layers of interspersed longitudinally arranged SMCs (l-SMCs) are shown in blue (see also Figure 12), and circumferential radial SMCs (r-SMCs) in orange and red; each of which contacted each other and often adjacent endothelial cells. Examples are indicated by r-MEJ, r-SMC to EC junction; l-MEJ, l-SMC to EC junction; l-l-MJ, l-SMC to l-SMC junction; r-r-MJ, r-SMC to r-SMC junction.

**Figure 12:**
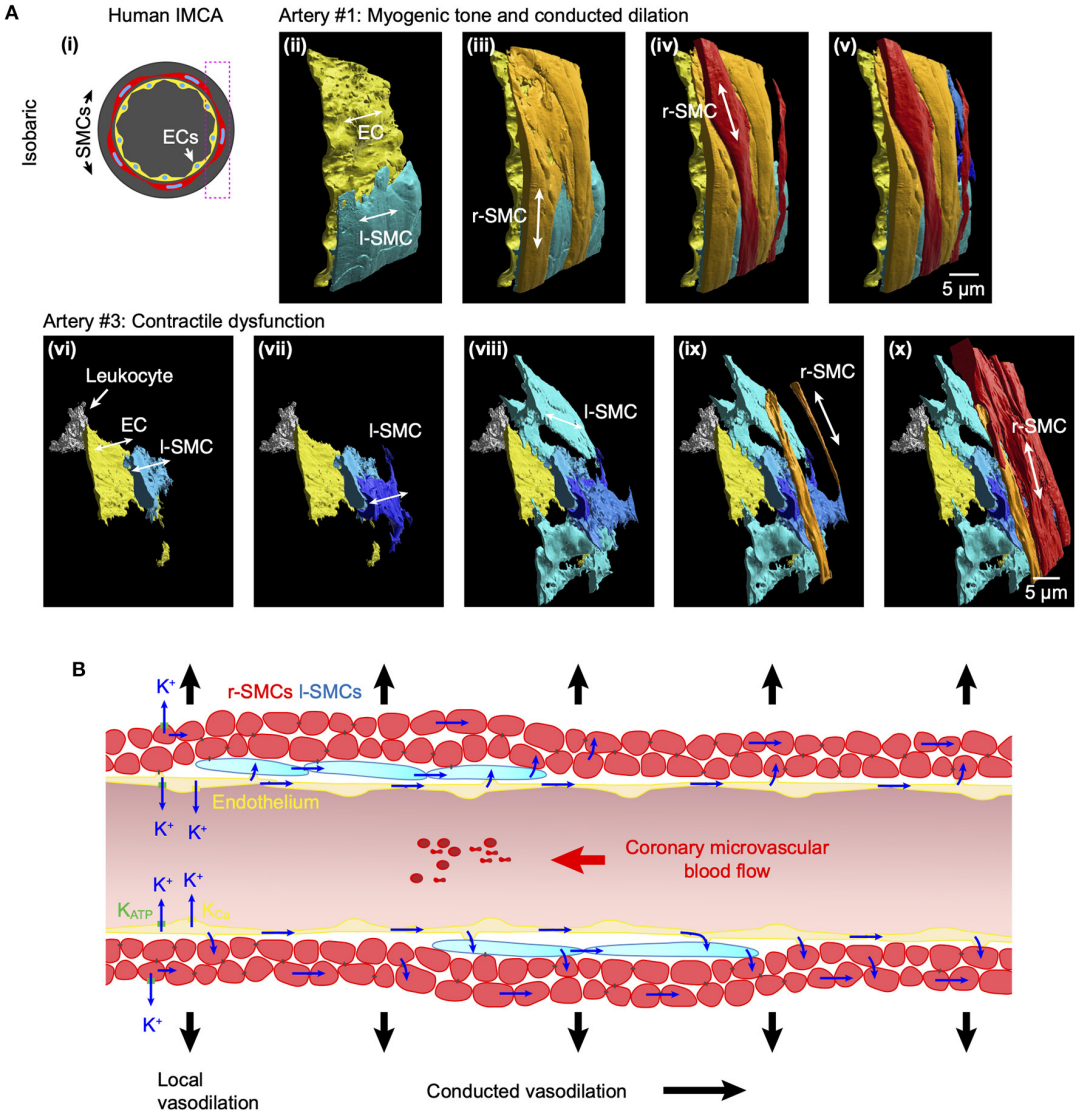
Arrangement of SMCs in human RA-IMCAs with and without contractile function. **(A)** Not all human IMCAs developed myogenic tone, but cell-cell contacts were always visible between ECs, r-SMCs and l-SMCs. Pressurized human IMCAs **(i)** were fixed and processed for SBF-SEM. Artery #1 is shown here from the rear, with sequence of added cell types **(ii–v)**. Artery #3 did not develop myogenic tone, had missing ECs and was fibrotic. Despite this, ECs made contact with l-SMCs, many of which made contact with each other and then ultimately the r-SMCs in the outer surface **(vi–x)**. An inflammatory cell (leukocyte) is attached to the endothelium. **(B)** Schematic of conduction pathways in coronary IMCAs. In human, porcine and rat IMCAs l-SMCs are found between the endothelium and circumferentially-orientated radial r-SMCs. The l-SMCs provide an additional pathway for intercellular communication and, where gap junctions are present, will facilitate conducted dilation.

The re-constructed 3D-structure of coronary arteries with contractile dysfunction showed that despite poor contractile function the ECs made multiple contacts with l-SMCs and r-SMCs, filling the gaps between the dense collagen and elastin present in the arterial wall (Figures 10–12). Artery #2 (Table 2) developed <10% MT, yet conducted dilation could still be observed in response to focally applied bradykinin (Figure 10). Artery #3 (Table 2) did not develop myogenic tone, hence conducted dilation could not be assessed. Nevertheless, it was striking that when cells were observed as single images in a xy-axis focal plane (Figure 11B), the cells did not appear to contact each other, yet when they were reconstructed in the z-axis, it was clear that each cell made multiple intercellular contacts, both homo- and heterocellular (Figures 10–12), highlighting the importance of this reconstruction in formulating the architecture of arteries.

## Discussion

The current study reveals a remarkably similar profile of vasodilation to bradykinin in myogenically-active human and porcine intramyocardial arteries. IMCAs were isolated from either atria or ventricle, with access to organ donor biopsies providing a rare opportunity to study human left ventricular IMCAs. The only notable difference between the human and porcine vessels was the potency of bradykinin. We also show importantly that myogenically contracting human and porcine IMCAs can develop robust conducted vasodilation following restricted application of either bradykinin or in porcine IMCAs to adenosine, and that the underlying cellular mechanism varies with each autacoid. Bradykinin generated hyperpolarization and vasodilation by activating EC K_Ca_ channels, with apparent no input from either NO or K_IR_ channels, while adenosine vasodilation relied on activation of both K_ATP_ and K_IR_ channels. Overall, our data support the ongoing use of right atrial appendage biopsies from patients and from pigs undergoing cardiopulmonary surgery as a useful model of human IMCAs.

Any dilator agonist or autacoid able to stimulate local vascular hyperpolarization will potentially also initiate conducted vasodilation (30, 31), depending on extensive gap-junction coupling, to allow hyperpolarization to spread axially along the arterial endothelium (31–33). The hyperpolarizing current spreads radially to the smooth muscle layers *via* heterocellular myoendothelial gap junctions, with the resulting hyperpolarization causing vasodilation by reducing the open-probability of voltage-gated calcium channels (8, 9). The spread of hyperpolarization along the endothelium appears to be facilitated, as the length constant is greater than expected for a purely passive process (34). Whether this reflects a difference in the duration of hyperpolarizing stimulus or an additional facilitating mechanism is not clear. However, one suggestion is that this facilitation is at least in part due to the activity of vascular K_IR_ channels (11), but there are likely other as yet undefined processes to consider.

The endothelium usually expresses two forms of K_Ca_ channel, of small (SK_Ca_) and intermediate (IK_Ca_) conductance, that are activated by any agonist/autacoid that increases EC cytoplasmic calcium. This distribution contrasts with the large conductance K_Ca_ channels (BK_Ca_ channels), usually confined to SMCs. Bradykinin-evoked vasodilation in human coronary arterioles preconstricted with endothelin appears to be entirely due to hyperpolarization generated by activation of K_Ca_ channels, without any significant contribution from either nitric oxide or cyclooxygenase derivatives (12). In both human and porcine coronary IMCAs this endothelium-dependent hyperpolarization may reflect the release of H_2_O_2_ and/or epoxyeicosatrienoic acids activating SMC BK_Ca_ channels (12, 35–37). However, in human coronary IMCAs preconstricted with the thromboxane-mimetic U46619, bradykinin-evoked vasorelaxation is due to hyperpolarization generated solely by EC SK_Ca_ and IK_Ca_ channel activity and by the release of NO (13). This finding was based on the ability of a specific SK_Ca_ blocker apamin combined with charybdotoxin (which blocks both intermediate- and large-conductance K_Ca_ channels, IK_Ca_ and BK_Ca_) and the NO scavenger hydroxocobalamin to markedly inhibit vasodilation. However, substituting charybdotoxin with a specific BK_Ca_ blocker, iberiotoxin failed to modify vasodilation (13). Our data were obtained in IMCAs during spontaneous myogenic constriction, with no need for agonist-evoked vasoconstriction. Although bradykinin evoked both local and conducted vasodilation through hyperpolarization, this was predominantly due to activation of EC SK_Ca_ and IK_Ca_ channels, as it was abolished either by raised extracellular potassium, or much reduced by the selective KCa blockers, apamin with TRAM-34, consistent with data from other groups (10, 12) (summarized in Table 3). In addition, we now show these K_Ca_ channel proteins are expressed and localized within both human and porcine IMCAs ECs, as in other species. Lack of inhibition with iberiotoxin indicates there is not a predominant role for BK_Ca_ in bradykinin vasodilation against myogenic tone, in arteries with myogenic tone matched to those of healthy pigs (18). Although these data contrast with previous observations, they may be reconciled by the finding that myoendothelial cell-cell contacts decline as microvascular dysfunction develops (18), perhaps secondary to large coronary artery disease (note the previous use of CABG surgery patients) (10, 12). In the latter studies the arteries may adapt to rely more on a diffusible endothelial factor acting to stimulate vasodilation via SMC BK_Ca_ channels. SK_Ca_ and IK_Ca_ together generate endothelium-dependent hyperpolarization, which is the primary endothelial vasodilator mechanism in many small arteries (9). Interestingly, some vasodilation did persist after block of EDH (combined block of SK_Ca_ and IK_Ca_ during NO synthesis block with L-NAME), but was abolished by raised extracellular K^+^, which may indicate input from an as yet unidentified potassium conductance. Whether this residual vasodilation to bradykinin in myogenically-active porcine and human atrial intra-pectinate arteries is in any way reliant on EC cytochrome P450 mono-oxygenase, and possibly BK_Ca_ channels, as suggested in ventricular subepicardial arterioles (35), remains to be established.

**Table 3:** Summary of some key published responses to bradykinin in human IMCAs.

**Source of arteries**	**Diameter (μm)**	**Pressure (mmHg)**	**Tone**	**Mechanism of dilation**	**References**
RA-Endo; LV-Endo/Epi	103 ± 2 (RA); 148 ± 10 (LV)	60	MT or ET-1	Not explored; LV data not shown	(38)
RA	97 ± 4	60	ET-1	BK_Ca_- and SK_Ca_-mediated hyperpolarisation	(12)
Branch off LAD	160–600; mean 380	100 (WM)	U46619	NO, K_IR_, Na^+^/ K^+^ ATPase; IK_Ca_ and SK_Ca_	(13)
RA	158 ± 6	60	ACh or ET-1	Superoxide and H_2_O_2_ production	(37)
RA	124 ± 8	80	MT	Conducted dilation: Gap junction-dependent, IK_Ca_ and SK_Ca_; NO in young (<64 y) subjects	(10)
Parietal pericardium	187 ± 4	100 (WM)	K^+^, U46619, ET-1	Agonist dependent; NO for K^+^ and U46619, H_2_O_2_ for ET-1	(39)
RA-Endo LV-Endo	153 ± 6	80	MT	EC Ca^2+^-dependent activation of IK_Ca_ and SK_Ca_	*

Endo, endocardial surface; Epi-epicardial surface; WM, wire myograph (isometric tension); MT, myogenic tone; ET-1, endothelin-1; ACh, acetylcholine; *, current study.

There has been only one previous report of conducted vasodilation in human RA coronary arteries during myogenic vasoconstriction and this also indicated a central role for SK_Ca_ and IK_Ca_ channels during bradykinin stimulation (10). Furthermore, this study showed that conducted vasodilation to bradykinin was abolished by the gap junction uncouplers, carbenoxolone or 18α-glycyrrhetinic acid, neither of which altered local vasodilation. Interestingly, NO was proposed to facilitate the passage of hyperpolarizing current along the endothelium, as the vasodilation 500 μm upstream from the bradykinin delivery pipette was sensitive to block of NO synthase even though K_Ca_ channels were still available (10). The authors also showed that conducted vasodilation was less in arteries from older (>64 years) compared to younger patients, suggesting an age-linked decrease in NO bioavailability might be responsible (10) (Table 3). This was the first time NO had been implicated directly in conducted vasodilation, as previous studies with skeletal muscle had discounted the possibility (40). Our data do not support a predominant role for NO in either local or conducted vasodilation in human or pig IMCAs. One possible explanation for these differences may relate to the type of patient recruited. Biopsies came from patients with established coronary artery disease (26 of 30 patients undergoing CABG surgery) in the study by Feher et al. (10) whereas only 3 CABG patients were included in our present study, and none of these biopsies were used to in experiments to measure conducted vasodilation. This patient-specific difference clearly warrants further investigation, as it has potential implications for the control of blood flow within the microcirculation of patients with epicardial coronary artery inflammation, intimal plaque formation and microvascular ischaemia. It also suggests that arterial function may be better preserved in patients undergoing valve surgery. By extension, our study supports the use of the porcine-derived samples as a model for healthy human arteries. Our high resolution microscopy also shows endothelial and smooth muscle cell contacts in RA-IMCAs, supporting the previous functional assessment using gap-junction uncouplers (10). Whether functional myoendothelial contacts remain in the arterioles from CABG surgery patients, and how NO might influence electrical coupling and current spread are clearly areas that merit further investigation. It seems unsurprising that with age and large coronary artery disease multiple mechanisms may contribute to disrupt coronary microvascular blood flow.

The conducted dilation to bradykinin was not affected by low micromolar Ba^2+^ in both human and porcine RA-IMCAs, arguing against a role for K_IR_ channels in either local or conducted vasodilation. Notably, Ba^2+^ visibly contracted each artery suggesting a functional activation of K_IR_ channels during myogenic tone. In contrast a previous study using porcine coronary arterioles (50–110 μm) suggested a significant functional role for K_IR_ channels in bradykinin-mediated vasodilation (11). These divergent observations may reflect the source for the arteries. We isolated intra-pectinate arteries, while the earlier study used branches of the left anterior descending and circumflex epicardial arteries (11, 41). Our data are in accordance with the concentration-dependent vasodilation to bradykinin in the ventricular biopsies, with an EC_50_ near 0.1 nM (11).

While responses to bradykinin are useful to show the importance of the endothelium, clinically the assessment of coronary flow reserve usually follows an infusion of adenosine, which serves as a good indicator of vasodilator capacity within the heart. Adenosine is usually released during coronary metabolic stress and stimulates coronary vasodilation *via* both endothelial and smooth muscle receptors. We compared the ability of adenosine to evoke conducted vasodilation to bradykinin. Our data confirm a role for glibenclamide-sensitive K_ATP_ channels in adenosine-evoked vasodilation of porcine coronary arterioles, channels that are expressed in both EC and SMC cells in subepicardial arterioles (29, 42–44). The endothelium-dependent component of vasodilation in porcine coronary arterioles has been shown to rely on NO release (29, 42, 43) *via* a pertussis toxin-sensitive pathway (42). This sensitivity suggests G_i/o_-coupling, and as both A_1_ and A_3_ receptors are G_i/o_ linked, either or both may be expressed on ECs and cause NO release. Furthermore, smooth muscle-dependent dilation to adenosine was fully blocked by glibenclamide (but not pertussis toxin) (42), supporting a direct role for the G_s_-coupled A_2A_ receptors causing vasodilation following hyperpolarization of SMCs (42). In contrast, studies in large porcine coronary arteries have failed to reduce adenosine-mediated vasodilation with glibenclamide (45). Furthermore, while adenosine evokes robust dilation in human RA-IMCAs studied *ex vivo* (46, 47), it was neither endothelium-dependent (47) nor sensitive to glibenclamide (46). This can be explained by variation in the signaling pathways downstream of the adenosine receptor subtypes across species and arteriolar diameters. Of relevance here is that other K^+^ channels have also been suggested to play a role in adenosine-mediated porcine coronary artery dilation, including K_IR_ channels (11) and K_v_7 channels (48). Clearly, the mechanisms that underly adenosine-mediated vasodilation in both the porcine and human coronary microvasculature requires further characterization.

An important consideration in the coronary microcirculation is the unusual presence of l-SMCs between the endothelium and r-SMCs. These cells were observed in human and porcine atrial and ventricular IMCAs (18) and now in rat septal intramuscular arteries. While the cells appear to have a synthetic phenotype in healthy arteries, they change to a more contractile phenotype in human RA-IMCAs with contractile dysfunction (18). The extensive homo- and heterocellular cell-cell contacts between ECs and l-SMCs and also from l-SMCs to r-SMCs in human RA-IMCAs with and without contractile dysfunction suggests a novel physiological role for l-SMCs: facilitating the spread of current and potentially chemical signals between cells, either *via* gap junctions or by the diffusion of released factors. While we cannot point to which pathways operate, there is evidence that gap junction uncouplers block conducted dilation in human RA-IMCAs (10), and that electrical coupling enables action potential-like spikes dependent on VGCCs in small arteries (49). Here we show that in rat septal arteries these depolarizing spikes (26) are associated with Ca^2+^ flashes that are synchronous between all the SMCs in a field of view, both l-SMCs and r-SMCs. This strongly supports electrical coupling as a signaling mechanism between the SMCs, and raises the intriguing possibility that l-SMCs support the crucial role of ECs in propagating signals upstream to evoke conducted dilation (32) in the coronary microcirculation (Figure 12B).

### Study limitations

A major limitation to understanding how the coronary microcirculation works is not being able to establish the contribution of conducted dilation to the control of coronary blood flow *in vivo*. While it has been shown that both bradykinin and adenosine are vasodilators in the coronary microcirculation *in vivo* (50), it is not possible to pinpoint the release or delivery of each agonist to assess the extent of electrical coupling in influencing upstream artery diameter. Furthermore, flow-induced dilation may contribute alongside conducted dilation, particularly in the larger diameter arteries. Therefore, responses must be studied under controlled conditions *ex vivo*. In the present study, a lack of tissue availability means we have been unable to establish whether conducted vasodilation is significant in human (or pig) ventricle-derived IMCAs. This aspect warrants future investigation. Future *ex vivo* studies should be extended to disease models, including porcine models of acute myocardial infarction and ischaemia and non-obstructive coronary artery disease, as steps toward understanding the operation and failing of the human coronary microcirculation (6).

## Conclusions

Our data demonstrate that intramyocardial arteries from the atria and ventricles of pigs and organ donors can be used for *ex vivo* studies of microvascular function. Obtaining access to viable ventricular biopsies is extremely difficult, so our *ex vivo* data showing responses to the EC-dependent dilator bradykinin have the same profile across species and heart chambers is an important step forward. Once vasodilation was initiated by focal application of bradykinin or adenosine in IMCAs, it spread with minimal decline along the entire length of these small coronary arteries, secondary to hyperpolarization.

## Data availability statement

The raw data supporting the conclusions of this article will be made available by the authors, without undue reservation.

## Ethics statement

The studies involving human participants were reviewed and approved by Oxford Research Ethics Committee. The patients/participants provided their written informed consent to participate in this study. The animal study was reviewed and approved by University of Bristol Research Ethics and University of Oxford Ethical Committee.

## Author contributions

KD conceived and designed the experiments, collected and analyzed data, prepared the figures, and wrote the manuscript. JL, LB, TB, and CG collected and analyzed data. KD and JL performed the statistical analysis. RA and MT provided human and porcine specimens, and contributed to manuscript preparation. KD, JL, LB, TB, MT, RA, and CG proof-read the manuscript. All authors contributed to the article and approved the submitted version.

## Funding

This work was supported by the British Heart Foundation (BHF) and Medical Research Council (MRC) Grants to RA [Grant Numbers BHF: PG/18/49/33833, IG/14/2/30991, PG/16/104/32652, and MRC MR/L012723/1] and by the Bristol NIHR Biomedical Research Center. Organ Donor Heart Collection was funded by the NIHR Cambridge/Newcastle Blood and Transplant Research Unit (BTRU). In addition, this work was supported by British Heart Foundation Grants to KD [Grant Numbers FS/08/033/25111, FS/13/16/30199, IG/13/5/30431, PG/18/11/33552, and PG/20/10260] and by the Oxford BHF Centre of Research Excellence [Grant Number RE/13/1/30181].

## Conflict of interest

The authors declare that the research was conducted in the absence of any commercial or financial relationships that could be construed as a potential conflict of interest.

## Publisher's note

All claims expressed in this article are solely those of the authors and do not necessarily represent those of their affiliated organizations, or those of the publisher, the editors and the reviewers. Any product that may be evaluated in this article, or claim that may be made by its manufacturer, is not guaranteed or endorsed by the publisher.

## Supplementary material

Supplementary Video 1 3D render of cells in the wall of an isolated, cannulated and pressurized human RA-IMCA human with myogenic tone and conducted dilation to bradykinin, Artery #1. Refer to Figures 2, 10A, 12A for details; ECs yellow, l-SMC shades of blue, r-SMCs shades of red/orange.

Supplementary Video 2 Local and conducted dilation to bradykinin in a human RA-IMCA. Top: Simultaneous transmitted and fluorescence images. Bottom: Fluorescence images showing the autofluorescence of the elastin along the artery, along with the delivery of bradykinin. The corresponding time course is shown in Figure 3.

Supplementary Video 3 Local and conducted dilation to bradykinin in a porcine RA-IMCA. Top: Simultaneous transmitted and fluorescence images. Bottom: Fluorescence images showing the delivery of bradykinin, the only visible fluorescence. The corresponding time course is shown in Figure 3.

Supplementary Video 4 3D render of cells in the wall of an isolated, cannulated and pressurized human RA-IMCA with no myogenic tone, Artery #3. Refer to Figures 10A, 11, 12A for details; ECs yellow, l-SMC shades of blue, r-SMCs shades of red/orange, leukocytes white.
